# Supplementary material for: Effectiveness of acupuncture therapy for the prevention of emergence agitation in children: A systematic review and meta-analysis with trial sequential analysis
Source: PLoS One. 2023 Jun 6;18(6):e0286790. doi: 10.1371/journal.pone.0286790 (PMC10243631; doi:10.1371/journal.pone.0286790)

**Supplemental Material**

**Effectiveness of acupuncture therapy for the prevention of emergence agitation in children: A systematic review and meta-analysis with trial sequential analysis**

***Supplemental Appendix 1: Search Strategy for PubMed***

|  | Search strategy for PubMed |
| --- | --- |
| Number | **Search terms** |
| #1 | (“acupuncture”[Mh] OR “acupuncture”[tiab] OR “acupuncture therapy”[Mh] OR “acupoint”[tiab] OR “acupotomy”[tiab] OR “moxibustion”[Mh] OR “moxibustion” [tiab] OR “acupressure”[Mh] OR “acupressure”[tiab] OR “electrical stimulation”[tiab] OR “electroacupuncture”[Mh] OR “electroacupuncture”[tiab] OR “electroacupuncturing”[tiab] OR “transcutaneous electric nerve stimulation”[Mh] OR “shiatsu”[tiab]) |
| #2 | (“child”[Mh] OR “child”[tiab] OR “children”[tiab] OR “pediatrics”[Mh] OR “pediatrics”[tiab] OR “pediatric”[tiab] OR “paediatrics”[tiab] OR “paediatric”[tiab] OR “infant”[Mh] OR “infant”[tiab] OR “adolescent”[Mh] OR “adolescent”[tiab]) |
| #3 | (“anesthesia” [Mh] OR “anesthesia” [tiab] OR “anaesthesia” [tiab] OR “perioperative period"[Mh] OR "perioperative care"[Mh] OR “perioperative” [tiab] OR “intraoperative” [tiab] OR “preoperative” [tiab] OR “postoperative” [tiab]) |
| #4 | #2 AND #3 |
| #5 | (randomized controlled trial[pt] OR controlled clinical trial[pt] OR randomized[tiab] OR placebo[tiab] OR drug therapy[sh] OR randomly[tiab] OR trial[tiab] OR groups[tiab] NOT (animals [mh] NOT humans [mh])) |

***Supplemental Fig 1***

A: Forest Plot showing Children’s Hospital of Eastern Ontario Pain Scale (CHEOPS) scores

B: Forest Plot showing time taken for extubation

C: Forest Plot showing post-anesthesia care unit (PACU) length of stay


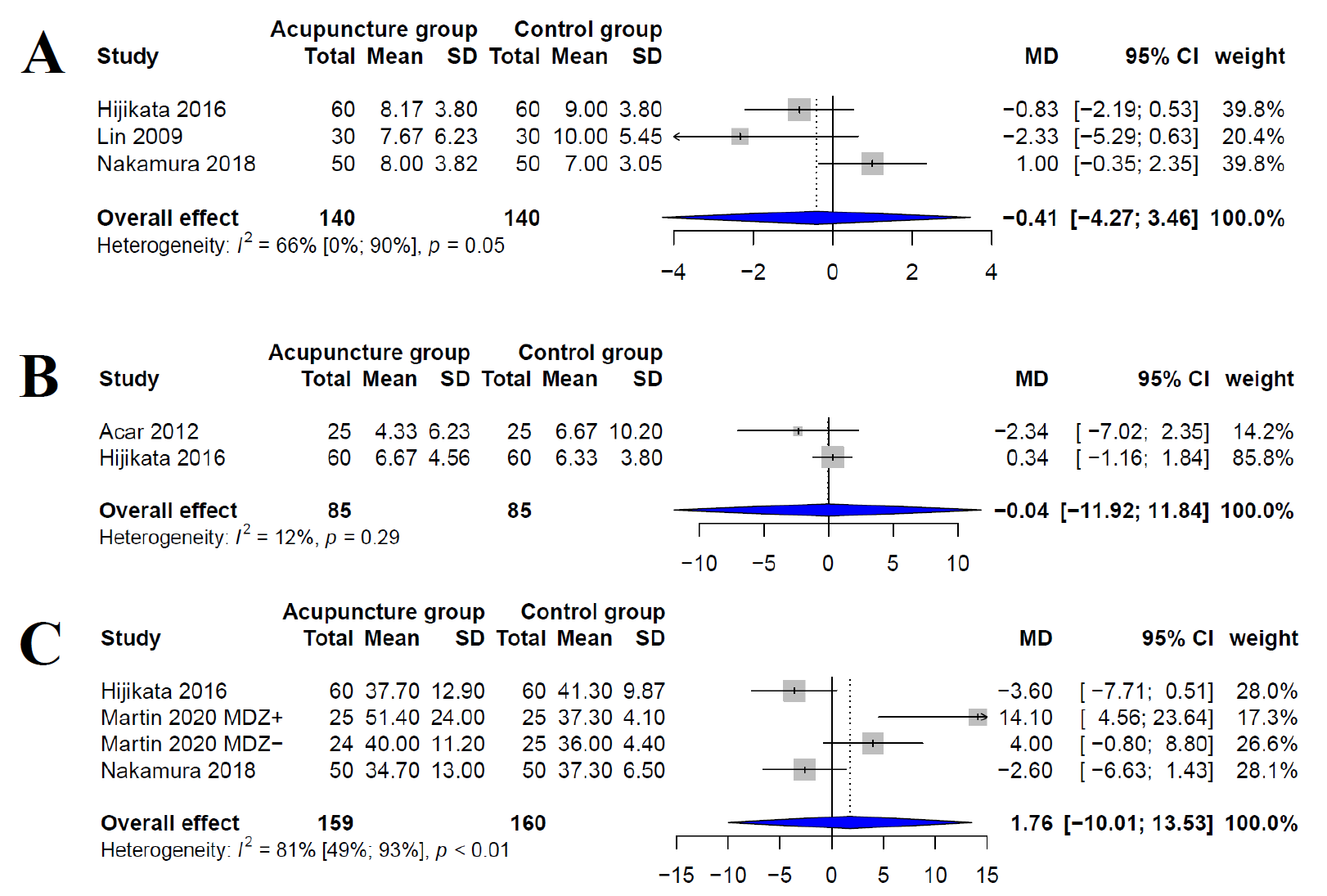


***Supplemental Fig 2 Forest Plot showing the risk of bias analysis***


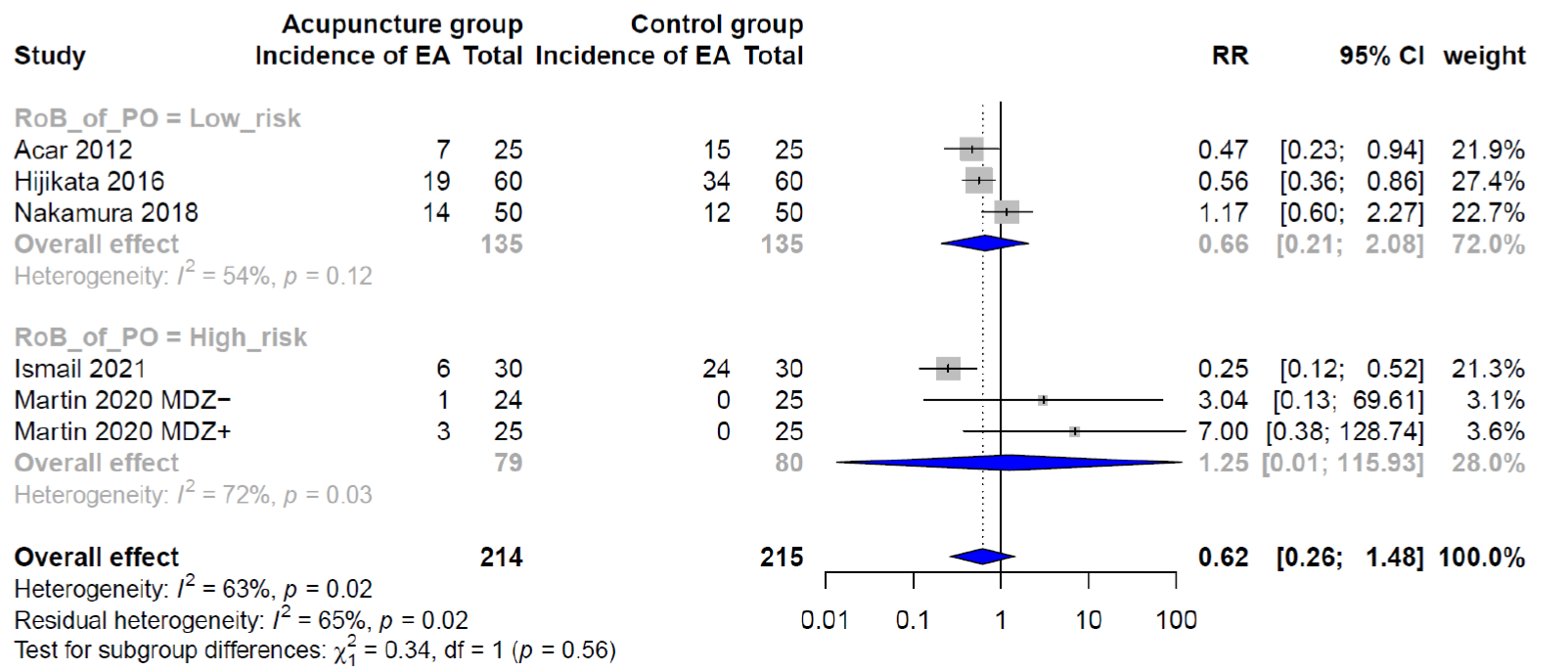


***Supplemental Fig 3 Forest Plot showing surgery type subgroup analysis***
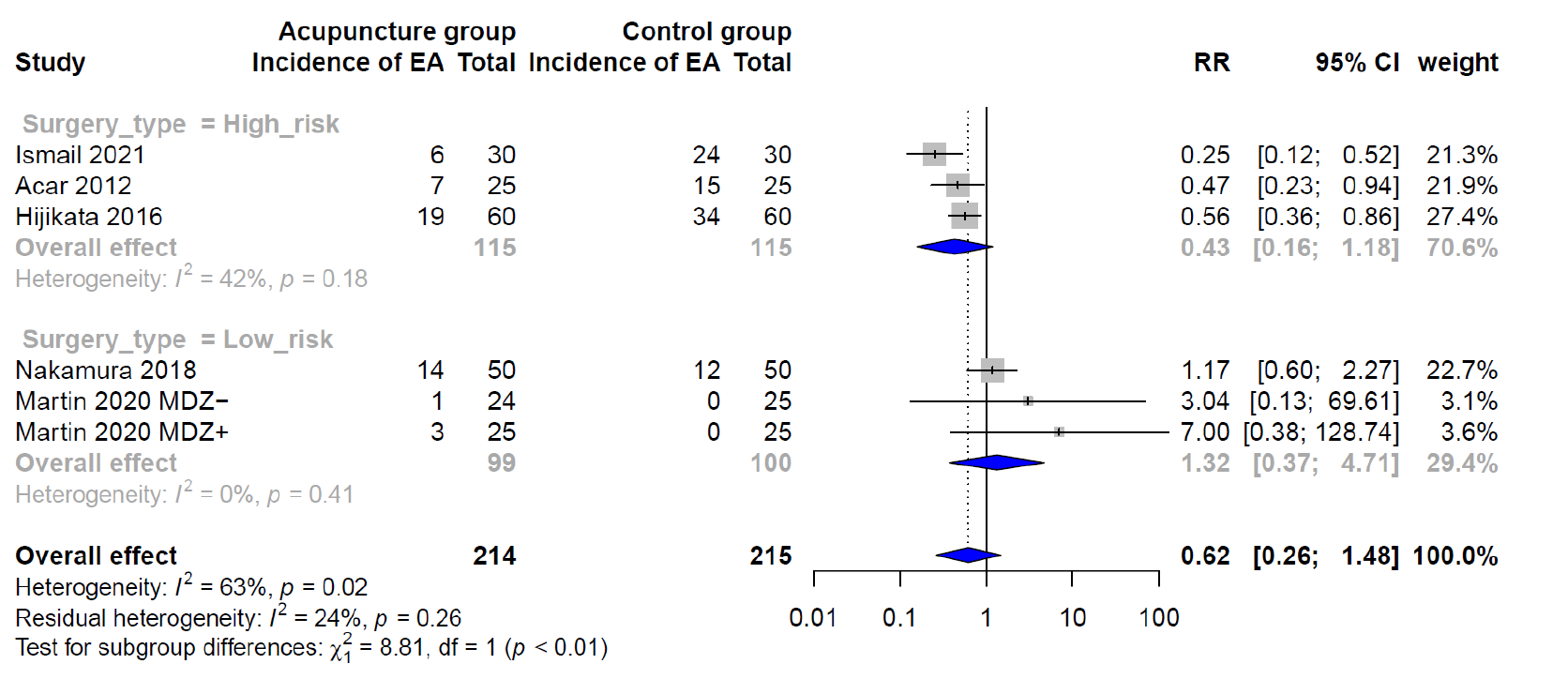


***Supplemental Fig 4 Forest Plot showing acupuncture type subgroup analysis***


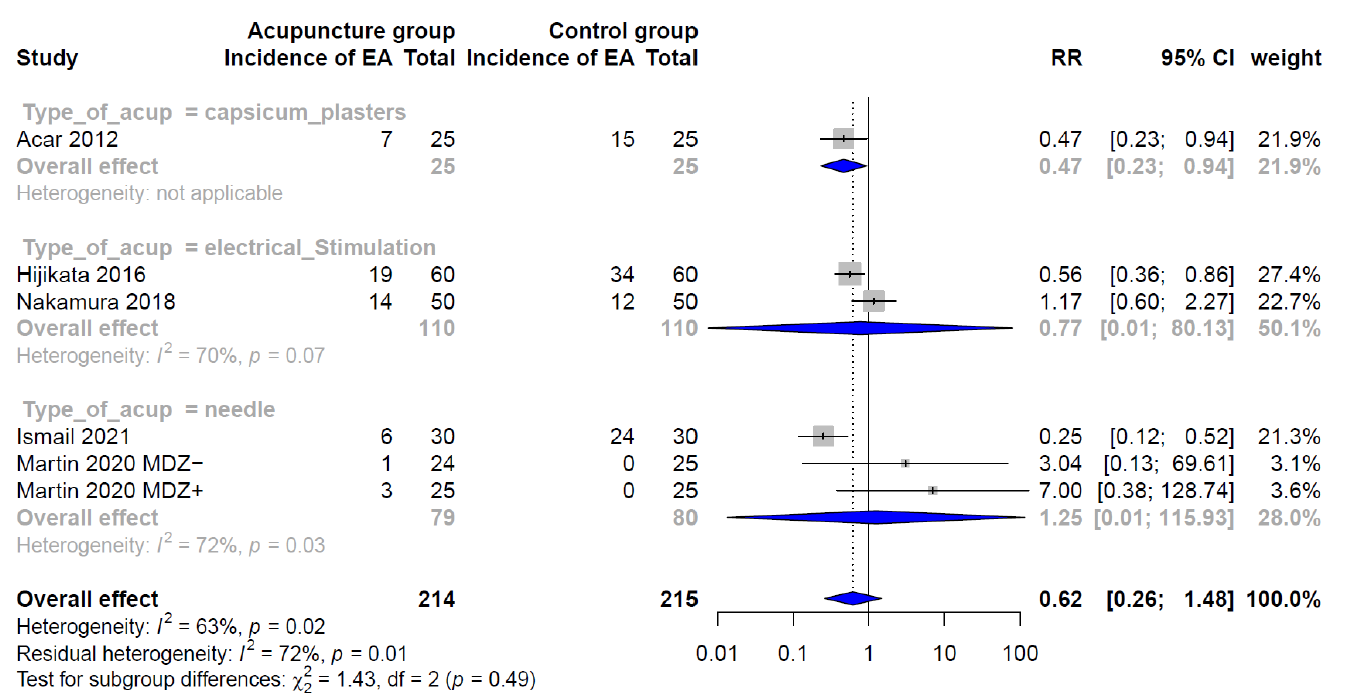


***Supplemental Fig 5 Forest Plot showing point selection subgroup analysis***

***Supplemental Fig 6 The risk of bias for secondary outcomes***


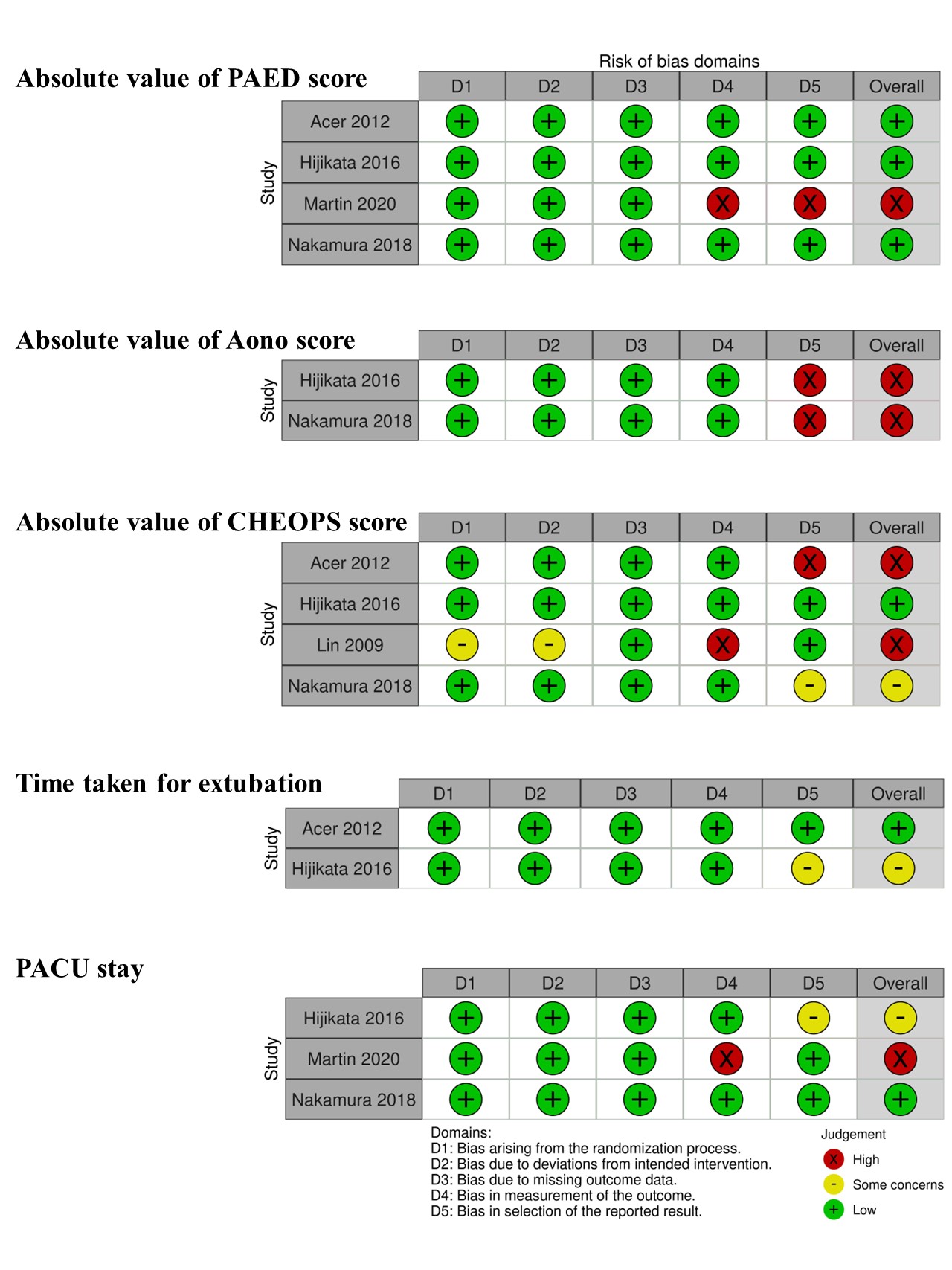

Supplement: S1 File — (DOCX) [file pone.0286790.s002.docx]
