# Supplementary material for: Effectiveness of acupuncture therapy for the prevention of emergence agitation in children: A systematic review and meta-analysis with trial sequential analysis
Source: PLoS One. 2023 Jun 6;18(6):e0286790. doi: 10.1371/journal.pone.0286790 (PMC10243631; doi:10.1371/journal.pone.0286790)
Supplement: Attachment — Submitted filename: Response_to_Reviewers_02.docx [file pone.0286790.s003.docx]

Dear Editor:

We sincerely appreciate your constructive feedback on our manuscript (PONE-D-22-28712). We have carefully considered your comments and made appropriate revisions to address your concerns. Our responses to each of your comments are detailed below. In this text, the editor’s and reviewers' comments are presented in black font, while our responses are in red font.

We trust that these revisions have improved our manuscript and addressed the concerns you raised. Thank you once again for your valuable feedback and for providing us with the opportunity to enhance our work.

Sincerely,

Takahiro Mihara

Department of Health Data Science, Yokohama City University Graduate School of Data Science, Yokohama City University

Yokohama 236-8511, Japan

Phone: +8145-787-2800

E-mail: meta.analysis.r@gmail.com

**Additional Editor Comments:**

Some details were not provided, e.g. the inclusion criteria of the review. The primary outcome was incidence of EA, however, the diagnostic criteria of EA was not specified. It's very important to identify how to determine EA according to the PACU scores. Besides, in Table 1 the type of control in each included trial should also be reported.

**Response:** Thank you for your feedback.

1. In the Methods section, we have now included detailed information regarding the inclusion criteria. This addition will provide readers with a clearer understanding of the parameters we used to select the studies for our analysis.

2. To further enhance the clarity and transparency of our work, we have specified the diagnostic criteria for Emergence Agitation (EA) employed in each study. This information has been incorporated into Table 1, allowing for easy comparison and assessment of the included studies.

3. Lastly, we recognize the importance of providing information about the type of control used in each study. To address this, we have added a new column, "Type of Control," to Table 1. This will enable readers to better understand the context of each study and facilitate comparisons across different control groups.

Purpose of this review is to assess the effectiveness of acupuncture therapy in preventing EA in children undergoing general anesthesia, and the current results showed no difference between groups. With limited number of included participants, results of subgroup analysis may not really suggest the conclusions in the text. I suggest that authors carefully evaluate the existing evidence and draw objective conclusions.

**Response:** Thank you for your comment. In light of the results from the subgroup analysis, we have tempered the strength of the conclusion in the Discussion section and removed it from the Conclusion.

**Reviewer #1**

I do not have any concerns about this paper and all of the criteria for publication have been met. This paper appropriately identifies the lack of evidence for and high quality studies of acupuncture for EA.

**Response:** Thank you for your positive comments.

**Reviewer #2**

This study is an important report for improving the quality of integrative medicine in recent years. In particular, acupuncture therapy is noninvasive and is expected to play a role as an adjunctive treatment. Thus, it is necessary to accumulate scientific evidence and information, and the authors have organized the data for acupuncture therapy in preventing emergence agitation. Although the data appear to be factual, there are a few questions.

**Response:** Thank you for your positive comments. Our point-by-point responses to your comments are below, and we hope that our replies correctly answer your questions.

The selected papers seem to be a collection of reports that chose the acupuncture point of HT7 (Shenmen). Why is HT7 regularly used in previous studies? Please describe in Background or Discussion.

**Response:** Thank you for your comments. We have added the following sentence to the Background section as per your suggestion:

“A potential acupoint for suppressing emergence agitation in pediatric anesthesia is HT7 (Shenmen). HT7 is recognized as an acupoint that promotes tranquility [14]. In animal studies, the therapeutic effects of HT7 acupuncture therapy on psychiatric and neurological disorders have been documented [15,16].”

Some are written in the limits (L300), acupuncture treatment focused on HT7 is just ineffective, other acupuncture points may be effective? Can you suggest other acupoints that are more effective than HT7 from an oriental medicine side?

**Response:** In the context of Oriental medicine, HT7 is considered an effective acupoint for preventing emergence agitation, as it is known to have a calming effect on mood and has demonstrated effectiveness in animal studies. Unfortunately, we are not aware of any other acupoints that we could recommend for this purpose.

Shouldn't the Conclusion be "acupuncture treatment focusing on HT7" instead of "effects of acupuncture"? Please reconsider your conclusions along with the contents of the excluded papers.

**Response:** Thank you for your comment. Although our study aims to cover all types of acupuncture treatments, we found that all the studies we included ultimately focused on HT7. As you pointed out, there may be other acupuncture points with therapeutic effects. In the revised manuscript, we have added the following sentence in the Limitations section to emphasize that our study results cannot be extrapolated to points other than HT7: “All studies in this research chose to stimulate HT7 as the acupoint. While this may have reduced clinical heterogeneity in terms of treatment, it is important to note that our study results cannot be extrapolated to the stimulation of other acupoints.”

This research and analysis will hopefully lead to a stronger and better culture of acupuncture.

**Response:** Thank you for your positive comments.
