# Supplementary material for: Effectiveness of acupuncture therapy for the prevention of emergence agitation in children: A systematic review and meta-analysis with trial sequential analysis
Source: PLoS One. 2023 Jun 6;18(6):e0286790. doi: 10.1371/journal.pone.0286790 (PMC10243631; doi:10.1371/journal.pone.0286790)
Supplement: Attachment — Submitted filename: Response_to_ReviewersR1_01.docx [file pone.0286790.s004.docx]

Dear Editor:

We sincerely appreciate your constructive feedback on our manuscript (PONE-D-22-28712R1). We have carefully considered your comments and made appropriate revisions to address your concerns. Our response to your comment is detailed below. In this text, the reviewers' comments are presented in black font, while our responses are in red font.

We trust that these revisions have improved our manuscript and addressed the concerns you raised. Thank you once again for your valuable feedback and for providing us with the opportunity to enhance our work.

Sincerely,

Takahiro Mihara

Department of Health Data Science, Yokohama City University Graduate School of Data Science, Yokohama City University

Yokohama 236-8511, Japan

Phone: +8145-787-2800

E-mail: meta.analysis.r@gmail.com

**Reviewer #3**

The points highlighted in previous review have been successfully addressed. However, I would like to raise one concern to improve the quality of this manuscript. please describe the ‘emergence agitation’ in more details in the introduction section.

**Response:** Response: Thank you for your comment. As you pointed out, we have added an explanation about emergence agitation in the first paragraph of the Introduction section (page 5, lines 49 to 54).
